# Identification of Hospitals That Care for a High Proportion of Patients With Social Risk Factors

**DOI:** 10.1001/jamahealthforum.2021.1323

**Published:** 2021-07-02

**Authors:** Rachael Matty, Rebekah Heckmann, Elizabeth George, Andrea Barbo Barthel, Lisa G. Suter, Joseph S. Ross, Susannah M. Bernheim

**Affiliations:** 1 Center for Outcomes Research and Evaluation, Yale–New Haven Health System, New Haven, Connecticut; 2 Boston University School of Public Health, Boston, Massachusetts; 3 Department of Emergency Medicine, Yale University School of Medicine, New Haven, Connecticut; 4 Frank H. Netter School of Medicine, Quinnipiac University, Hamden, Connecticut; 5 Section of Rheumatology, Department of Medicine, Yale University School of Medicine, New Haven, Connecticut; 6 Department of Medicine, Division of Rheumatology, Veterans Affairs Connecticut Health System, New Haven, Connecticut; 7 Division of General Internal Medicine, Department of Medicine, Yale University School of Medicine, New Haven, Connecticut; 8 Yale University School of Public Health, New Haven, Connecticut

## Abstract

**Importance:**

Hospitals can face significant clinical and financial challenges in caring for patients with social risk factors. Currently the Hospital Readmission Reduction Program stratifies hospitals by proportion of patients eligible for both Medicare and Medicaid when calculating payment penalties to account for the patient population. However, additional social risk factors should be considered.

**Objective:**

To evaluate 7 different definitions of social risk and understand the degree to which differing definitions identify the same hospitals caring for a high proportion of patients with social risk factors.

**Design, Setting, and Participants:**

Across 18 publicly reported Centers for Medicare & Medicaid Services (CMS) hospital performance measures, highly disadvantaged hospitals were identified by the the proportion of patients with social risk factors using the following 7 commonly used definitions of social risk: living below the US poverty line, educational attainment of less than high school, unemployment, living in a crowded household, African American race (as a proxy for the social risk factor of exposure to racism), Medicaid coverage, and Agency for Healthcare Research and Quality index of socioeconomic status score. In this cross-sectional study, social risk factors were evaluated by measure because hospitals may serve a disadvantaged patient population for one measure but not another. Data were collected from April 1, 2014, to June 30, 2017, and analyzed from July 25, 2019, to April 25, 2021.

**Main Outcomes and Measures:**

The proportion of hospitals identified as caring for patients with social risk factors using 7 definitions of social risk, across 18 publicly reported CMS hospital performance measures.

**Results:**

Among 4465 hospitals, a mean of 31.0% (range, 28.9%-32.3%) were identified at least once when using the 7 definitions of social risk as caring for a high proportion of patients with social risk factors. Among all hospitals meeting at least 1 definition of social risk, a mean of 0.7% (range, 0%-1.0%) were identified as highly disadvantaged by all 7 definitions. Among hospitals meeting at least 1 definition of social risk, a mean of 2.7% (range, 1.3%-5.1%) were identified by 6 definitions; 6.5% (range, 5.9%-7.1%), by 5 definitions; 10.4% (range, 9.5%-12.1%), by 4 definitions; 13.2% (range, 10.1%-14.4%), by 3 definitions; 21.4% (range, 20.1%-22.4%), by 2 definitions; and 45.2% (range, 42.6%-47.1%), by only 1 definition. This pattern was consistent across all 18 performance measures.

**Conclusions and Relevance:**

In this cross-sectional study, there were inconsistencies in the identification of hospitals caring for disadvantaged populations using different definitions of social risk factors. Without consensus on how to define disadvantaged hospitals, policies to support such hospitals may be applied inconsistently.

## Key Points

**Question:**

Are hospitals that care for a high proportion of patients with social risk factors consistently identified using varied definitions of these factors?

**Findings:**

In this cross-sectional study, among 4465 US hospitals qualified for Centers for Medicare & Medicaid Services hospital performance measures, one-third were identified as caring for a high proportion of patients with social risk factors across 7 definitions of social risk; fewer than 1% met all 7 definitions. Most hospitals serving patients with social risk factors could be identified using 3 or 4 definitions.

**Meaning:**

Inconsistencies in identifying hospitals caring for high proportions of patients with social risk factors suggest value in developing a common definition of social risk.

## Teaser

This cross-sectional study determines whether different risk factors identify the same highly disadvantaged hospitals caring for a high proportion of disadvantaged patients using 7 definitions of social risk.

## Introduction

The 2016 21st Century Cures Act states that hospitals assessed by Centers for Medicare & Medicaid Services (CMS) outcome and payment measures are assigned to groups to allow for separate comparison of hospitals by their overall proportion of patients eligible for both Medicare and Medicaid (dual eligibility).^1^ This peer-group stratification of hospitals is used to assign a payment penalty threshold that is different for each group of hospitals. Using dual eligibility to group hospitals may address concerns that pay-for-performance programs do not account for social risk. Consideration of the social risk profile of a provider’s patients to classify providers in pay-for-performance programs will account for factors such as patient social support and functional status that may affect patient outcomes, hospital performance, and the resulting financial penalties.^2^ By financially penalizing hospitals without accounting for these social risk factors, there is a chance that providers caring for patients with social risk who are penalized will have fewer needed resources for improving quality, potentially accelerating disparities.^3^ However, a challenge in incorporating social risk into quality and payment measures is that no consensus among experts exists as to which social risk factors are best for identifying and stratifying providers.

Hospital-level indices for social risk are a valuable way to assess the population that a hospital serves. Such approaches are currently used for a variety of purposes. They are used by researchers to identify safety net hospitals to understand quality and safety, financial resources, and disparities. As noted above, they are also used to identify hospitals to assess performance and target resources.^2^ The Office of the Assistant Secretary for Planning and Evaluation supported dual eligibility as “the most powerful predictor of poor outcomes.”^4^^(p8)^ Although dual eligibility may include a high proportion of racial minority populations and low-income patients, it is not intended to be used as a composite measure for all social risk factors. For example, dual eligibility may not indicate someone’s educational attainment or whether they are living in a crowded household. Furthermore, dual eligibility is affected by state-to-state variations in Medicaid eligibility and enrollment, thus reducing ability to compare hospital service of disadvantaged populations between states. Experts have flagged additional social risk factors. The National Quality Forum^5^ prioritized social risk factors, including income, educational attainment, race, primary language, and patient living environment, for potential use in measuring risk adjustment or stratification. The National Academy of Medicine identified socioeconomic position; race, ethnicity, and cultural context; sex; social relationships; and residential and community context as social risk factors that affect health outcomes.^6^ The objective of this study was to evaluate 7 different definitions of social risk to understand the degree to which differing definitions identify the same hospitals.

## Methods

This cross-sectional study followed the Strengthening the Reporting of Observational Studies in Epidemiology (STROBE) reporting guideline. This work received Yale University Institutional Review Board approval under a protocol for research on CMS data. The Human Investigation Committee at Yale University approved an exemption for this study to use CMS claims and enrollment data and waived the requirement for informed consent because the research involved no more than minimal risk and could not be practicably performed without the waiver.

This cross-sectional study included US hospitals that publicly reported 1 or more of the 18 CMS 2018 hospital readmission, mortality, complication, and payment measures, which include data from April 1, 2014, to June 30, 2017. For each measure, highly disadvantaged hospitals were identified as those caring for a high proportion of patients with social risk factors. Highly disadvantaged hospitals were compared using 7 different definitions of social risk. A social risk factor was deemed useful to test based on (1) established face and/or empirical validity as a measure of social risk (eg, shown to be associated with greater risk of care disparities and/or worse patient outcomes) and (2) availability in CMS administrative data or publicly available data to ensure universal assessment of patient social risk. These definitions included living below the US poverty line, educational attainment below high school, unemployment, living in a crowded household, African American race (included as a proxy for the social risk factor of exposure to racism),^7^ Medicaid coverage, and Agency for Healthcare Research and Quality (AHRQ) index of socioeconomic status (SES) score.^8^ The first 6 social risk factors were chosen based on their relevance in other research and availability in national data sources.^5,6^ The AHRQ index of SES score was chosen because it is a weighted composite score of the first 4 social risk factors. For all the social risk factors, except for race, a patient is considered to have a social risk if they are in the 75th percentile (25th for AHRQ index of SES) on a continuous scale. For example, to identify patients with a low AHRQ index of SES, the 25th percentile of the national distribution of AHRQ index of SES scores was used. Patients with scores below the 25th percentile were considered to have low AHRQ index of SES.

To calculate a hospital’s proportion of patients who live below the US poverty line, possess educational attainment below high school, are unemployed, and live in crowded households, data from the American Community Survey 2013-2017 5-year estimate, as well as Medicare Part A Inpatient Claims (2016), were used. The proportion of African American patients treated at a hospital was calculated using data from Medicare Part A Inpatient Claims, which captures patient self-reported race. The proportion of Medicaid patients for each hospital was calculated using data from the American Hospital Association Survey from 2016.^9^ The AHRQ index of SES score used data from both the American Community Survey and Medicare Part A claims. The American Community Survey data used zip code–level data for calculating risk factors for each patient, whereas Medicare Part A Inpatient Claims uses patient-level data to identify social risk factors for the hospital population (Table 1).

**Table 1.** Definitions and Data Sources for Social Risk Factors

Social risk factor	Criteria for identification of patients with social risk factors	Source
Below US poverty line	Patients from zip codes where >18.8% of the residents are below the US poverty line	ACS 2013-2017 5-y estimate
		Medicare Part A Inpatient Claims 2016
Educational attainment below high school	Patients from zip codes where >16.1% of the residents aged ≥25 y have <12th grade education	ACS 2013-2017 5-y estimate
		Medicare Part A Inpatient Claims 2016
Unemployment	Patients from zip codes where >8.7% of the residents aged ≥16 y in labor force are unemployed and actively seeking work	ACS 2013-2017 5-y estimate
		Medicare Part A Inpatient Claims 2016
Crowded household	Patients from zip codes where >3.2% of the residents live in households containing ≥1 person per room	ACS 2013-2017 5-y estimate
		Medicare Part A Inpatient Claims 2016
African American race	Patients who self-identify as African American race	Medicare Part A Inpatient Claims 2016
Medicaid	Patients with Medicaid coverage	AHA Survey 2016^9^
AHRQ index of SES score	Patients from zip codes with an AHRQ index of SES score <48.1	AHRQ index of SES^8^
		ACS 2013-2017 5-y estimate
		Medicare Part A Inpatient Claims 2016

Abbreviations: ACS, American Community Survey; AHA, American Hospital Association; AHRQ, Agency for Healthcare Research and Quality; SES, socioeconomic status.

Hospitals were evaluated as serving a high proportion of patients with social risk factors by each measure because the patient population and number of hospitals included in the calculation differed by quality and payment measure. Therefore, a hospital may serve a high proportion of patients for a given social risk factor for one measure but not another. To identify a hospital as highly disadvantaged for a given social risk factor, the proportion of patients with each of the social risk factors was calculated for each hospital. For each measure, hospitals with fewer than 25 admissions were excluded because there is not enough information to calculate hospital performance, and therefore these hospitals are not included in payment programs (for that measure). Hospitals were ranked from the lowest proportion of patients with the social risk factor of interest to the highest proportion within each measure. Hospitals that ranked in the top decile for each measure were considered to have a high proportion of patients for that social risk factor. This approach was used to account for varying numbers of hospitals reporting each measure. Then the number of definitions that identified a given hospital as having a high proportion of patients with social risk factors (0-7 social risk factor definitions) was counted. Hospitals were categorized as highly disadvantaged if their patient population was identified in the top decile of 1 or more social risk factors. Of those highly disadvantaged hospitals, the percentage of hospitals identified by only 1 through all 7 social risk factors for each outcome and payment measure was calculated and summarized as a mean across measures. This process was repeated for each of the 7 social risk factors to understand how hospitals are identified with various social risk factors across multiple measures.

An additional analysis was completed to understand whether any combination of risk factors could account for most hospitals that were identified as caring for a high proportion of patients with social risk factors by at least 1 factor. For each measure, the combination of 3 and the combination of 4 risk factors that identified the most hospitals serving patients with social risk factors were calculated. This study consisted solely of descriptive statistics (ie, frequencies and percentages) and therefore did not include statistical tests as part of the analysis. Data were collected from April 1, 2014, to June 30, 2017, and analyzed from July 25, 2019, to April 25, 2021.

## Results

In the US, 4465 hospitals were evaluated using one of the CMS mortality, readmission, complication, or payment measures. Almost one-third (mean, 31.0% [range, 28.9%-32.3%] across measures) of all hospitals were identified as highly disadvantaged (Table 2). Of these hospitals, almost one-half (mean, 45.2% [range, 42.6%-47.1%] across measures) were identified by only 1 risk factor. As the number of social risk factors used to identify highly disadvantaged hospitals increased, fewer hospitals were identified. When the mean was taken across measures, among the hospitals meeting at least 1 definition of social risk, a mean of 0.7% (range, 0%-1.0%) of hospitals were identified by all 7 definitions; 2.7% (range, 1.3%-5.1%), by 6 definitions; 6.5% (range, 5.9%-7.1%), by 5 definitions; 10.4% (range, 9.5%-12.1%), by 4 definitions; 13.2% (range, 10.1%-14.4%), by 3 definitions; and 21.4% (range, 20.1%-22.4%), by 2 definitions.

**Table 2.** Identification of Hospitals Caring for a High Proportion of Patients With Social Risk Factors Across CMS Hospital Outcome and Payment Measures

Outcome or measure by condition	No. of hospitals in measure	No. of social risk factors identified^a^							
		≥1	1	2	3	4	5	6	7
Acute myocardial infarction									
Mortality	2427	702 (28.9)	299 (42.6)	154 (21.9)	92 (13.1)	73 (10.4)	46 (6.6)	31 (4.4)	7 (1.0)
Payment	2283	690 (30.2)	295 (42.8)	152 (22.0)	95 (13.8)	67 (9.7)	44 (6.4)	32 (4.6)	5 (0.7)
Readmission	2211	646 (29.2)	282 (43.7)	142 (22.0)	78 (12.1)	68 (10.5)	39 (6.0)	33 (5.1)	4 (0.6)
Coronary artery bypass graft									
Mortality	1017	316 (31.1)	146 (46.2)	65 (20.6)	32 (10.1)	38 (12.0)	22 (7.0)	13 (4.1)	0
Readmission	1013	314 (31.0)	145 (46.2)	63 (20.1)	35 (11.1)	38 (12.1)	20 (6.4)	13 (4.1)	0
Chronic obstructive pulmonary disease									
Mortality	3588	1149 (32.0)	528 (46.0)	251 (21.8)	154 (13.4)	117 (10.2)	71 (6.2)	19 (1.7)	9 (0.8)
Readmission	3645	1162 (31.9)	529 (45.5)	260 (22.4)	153 (13.2)	120 (10.3)	73 (6.3)	17 (1.5)	10 (0.9)
Heart failure									
Mortality	3693	1114 (30.2)	501 (45.0)	238 (21.4)	147 (13.2)	118 (10.6)	79 (7.1)	21 (1.9)	10 (0.9)
Payment	3513	1093 (31.1)	488 (44.6)	235 (21.5)	145 (13.3)	117 (10.7)	78 (7.1)	19 (1.7)	11 (1.0)
Readmission	3761	1138 (30.3)	514 (45.2)	246 (21.6)	148 (13.0)	123 (10.8)	76 (6.7)	21 (1.8)	10 (0.9)
Hip/knee									
Complication	2748	886 (32.2)	417 (47.1)	181 (20.4)	118 (13.3)	84 (9.5)	62 (7.0)	23 (2.6)	1 (0.1)
Readmission	2779	896 (32.2)	422 (47.1)	183 (20.4)	119 (13.3)	89 (9.9)	60 (6.7)	23 (2.6)	0
Hospital-wide readmission	4465	1440 (32.3)	670 (46.5)	302 (21.0)	206 (14.3)	143 (9.9)	90 (6.3)	19 (1.3)	10 (0.7)
Pneumonia									
Mortality	4237	1319 (31.1)	608 (46.1)	288 (21.8)	186 (14.1)	132 (10.0)	78 (5.9)	17 (1.3)	10 (0.8)
Payment	4076	1307 (32.1)	602 (46.1)	284 (21.7)	185 (14.2)	131 (10.0)	78 (6.0)	17 (1.3)	10 (0.8)
Readmission	4227	1314 (31.1)	605 (46.0)	286 (21.8)	189 (14.4)	128 (9.7)	79 (6.0)	17 (1.3)	10 (0.8)
Stroke									
Mortality	2590	794 (30.7)	349 (44.0)	168 (21.2)	108 (13.6)	86 (10.8)	49 (6.2)	27 (3.4)	7 (0.9)
Readmission	2527	764 (30.2)	330 (43.2)	165 (21.6)	102 (13.4)	82 (10.7)	50 (6.5)	28 (3.7)	7 (0.9)
Mean across measures, %	3044	31.0	45.2	21.4	13.2	10.4	6.5	2.7	0.7

Abbreviation: CMS, Centers for Medicare & Medicaid Services.

^a^ Unless otherwise indicated, data are expressed as number (percentage) of hospitals.

When evaluating combinations of risk factors that could account for most hospitals that were identified as caring for a high proportion of patients with social risk factors (Table 3), a mean of 74.9% (range, 73.0%-77.2%) of hospitals across measures could be identified by a combination of 3 risk factors, and a mean of 87.7% (range, 85.7%-89.6%) of hospitals could be identified by a combination of 4 risk factors. The combination of 3 risk factors that was most common for identifying the greatest proportion of hospitals identified by at least 1 social risk factor consisted of African American race, low educational attainment, and Medicaid coverage. The combination of 4 risk factors that was most common for identifying the greatest proportion of hospitals identified by at least 1 social risk factor was African American race, AHRQ index of SES score, living in a crowded household, and Medicaid coverage. Living below the poverty line was the only other factor that was included as part of a 3– or 4–risk factor combination.

**Table 3.** Combinations of Risk Factors That Account for Most Hospitals Caring for a High Proportion of Patients With Social Risk Factors

CMS hospital outcome or payment measure	No. identified by ≥1 social risk factor	Risk factors that identify the most hospitals serving patients with social risk factors			
		Combination of 3		Combination of 4	
		Risk factors	No. (%)	Risk factors	No. (%)
Acute myocardial infarction					
Mortality	702	African American race, AHRQ index of SES, and Medicaid or African American race, educational attainment, and Medicaid	542 (77.2)	African American race, AHRQ index of SES, crowded household, and Medicaid	628 (89.5)
Payment	690	African American race, AHRQ index of SES, and Medicaid	533 (77.2)	African American race, AHRQ index of SES, crowded household, and Medicaid	618 (89.6)
Readmission	646	African American race, educational attainment, and Medicaid	497 (76.9)	African American race, AHRQ index of SES, crowded household, and Medicaid	576 (89.2)
Coronary artery bypass grafting					
Mortality	316	African American race, crowded household, and Medicaid	239 (75.6)	African American race, AHRQ index of SES, crowded household, and Medicaid	278 (88.0)
Readmission	314	African American race, crowded household, and Medicaid or African American race, educational attainment, and Medicaid	238 (75.8)	African American race, AHRQ index of SES, crowded household, and Medicaid or African American race, crowded household, poverty, and Medicaid	275 (87.6)
Chronic obstructive pulmonary disease					
Mortality	1149	African American race, educational attainment, and Medicaid	849 (73.9)	African American race, AHRQ index of SES, crowded household, and Medicaid	988 (86.0)
Readmission	1162	African American race, educational attainment, and Medicaid	862 (74.2)	African American race, AHRQ index of SES, crowded household, and Medicaid	1000 (86.1)
Heart failure					
Mortality	1114	African American race, educational attainment, and Medicaid	842 (75.6)	African American race, AHRQ index of SES, crowded household, and Medicaid	979 (87.9)
Payment	1093	African American race, educational attainment, and Medicaid	830 (75.9)	African American race, AHRQ index of SES, crowded household, and Medicaid	963 (88.1)
Readmission	1138	African American race, educational attainment, and Medicaid	853 (75.0)	African American race, AHRQ index of SES, crowded household, and Medicaid	992 (87.2)
Hip/knee					
Complication	886	African American race, crowded household, and Medicaid or African American race, educational attainment, and Medicaid	653 (73.7)	African American race, AHRQ index of SES, crowded household, and Medicaid or African American race, crowded household, Medicaid, and educational attainment	761 (85.9)
Readmission	896	African American race, educational attainment, and Medicaid	662 (73.9)	African American race, crowded household, Medicaid, and educational attainment	771 (86.0)
Hospital-wide readmission	1440	African American race, educational attainment, and Medicaid	1056 (73.3)	African American race, crowded household, Medicaid, and educational attainment	1237 (85.9)
Pneumonia					
Mortality	1319	African American race, educational attainment, and Medicaid	963 (73.0)	African American race, AHRQ index of SES, crowded household, and Medicaid	1130 (85.7)
Payment	1307	African American race, educational attainment, and Medicaid	956 (73.1)	African American race, AHRQ index of SES, crowded household, and Medicaid	1122 (85.8)
Readmission	1314	African American race, educational attainment, and Medicaid	962 (73.2)	African American race, AHRQ index of SES, crowded household, and Medicaid	1127 (85.8)
Stroke					
Mortality	794	African American race, educational attainment, and Medicaid	607 (76.4)	African American race, AHRQ index of SES, crowded household, and Medicaid	707 (89.0)
Readmission	764	African American race, educational attainment, and Medicaid	587 (76.8)	African American race, AHRQ index of SES, crowded household, and Medicaid	684 (89.5)
Most common combination and mean across measures	NA	African American race, educational attainment, and Medicaid	74.9	African American race, AHRQ index of SES, crowded household, and Medicaid	87.7

Abbreviations: AHRQ, Agency for Healthcare Research and Quality; CMS, Centers for Medicare & Medicaid Services; NA, not applicable; SES, socioeconomic status.

## Discussion

In this cross-sectional study, approximately one-third of hospitals were identified as highly disadvantaged hospitals by at least 1 definition of social risk. Almost one-half of hospitals were only identified by 1 social risk definition, and less than 1% were identified by all 7 definitions. This supports our hypothesis that each definition identifies a different set of hospitals. These findings are critical to the discussion about how to address social risk in the CMS pay-for-performance programs. Recent legislation advises CMS to stratify hospitals for payment penalties by their proportion of patients with dual eligibility. However, our results suggest that a single definition of social risk may miss hospitals with significant populations of at-risk patients. This may limit the hospitals that are helped by this change in policy.

The findings of this study show that there is no single best definition of social risk that can be used to identify hospitals serving disadvantaged populations, but rather each definition of social risk identifies a different group of hospitals. These social risk factors can represent important different vulnerabilities and may be valuable to use in different contexts. Therefore, the best definition of social risk is highly dependent on the application of the social risk index, be it for research or payment policy. Using a combination of 3 or 4 risk factors may allow a more robust and parsimonious identification of hospitals serving high proportions of patients with social risk factors.

### Limitations

A limitation of this study is that the top decile of hospitals for any definition of social risk was used to identify hospitals with a high proportion of patients for that social risk factor. If this criterion were relaxed, more hospitals would be identified as serving high proportions of patients with social risk factors, and therefore slightly more hospitals would be identified by multiple social risk factors. However, using deciles instead of quintiles or quartiles ensured consistent identification of hospitals truly caring for a disproportionate share of patients with social risk. In addition, there are other social risk factors that could be considered to identify hospitals that serve a high proportion of patients with social risk factors; however, this study used only social risk factors that were available as part of the existing data that met our criteria.

## Conclusions

The findings of this cross-sectional study reveal variability in identifying hospitals caring for a disproportionate share of patients with social risk factors, supporting the need for continued research in this area. The use of multiple definitions of social risk may provide better insight into health care disparities than any individual definition of social risk.
